# Mammalian herbivory indirectly shapes savanna arthropod communities but only at very low or high levels

**DOI:** 10.1111/1365-2656.70221

**Published:** 2026-01-28

**Authors:** Bjoern Erik Matthies, Nicola Stevens, Jane K. Hill, Bosco Leturuka, Margaret Njuguna, Lucy K. Smyth, Matthew S. Rogan, Catherine W. Machungo, Jonathan E. M. Baillie, Jafford N. Rithaa, Catherine L. Parr

**Affiliations:** ^1^ Department of Earth, Ocean and Ecological Sciences, School of Environmental Sciences University of Liverpool Liverpool UK; ^2^ School of Geography and the Environment, Environmental Change Institute University of Oxford Oxford UK; ^3^ Natural State Isiolo Kenya; ^4^ Faculty of Agriculture and Environmental Studies Chuka University Chuka Kenya; ^5^ Department of Biology, Leverhulme Centre for Anthropocene Biodiversity University of York York UK

**Keywords:** ants, herbivory suppression, Kenya, livestock, semi‐arid savanna, woody encroachment

## Abstract

1. Savanna ecosystems support unique biodiversity and provide livelihoods for millions of people. Yet, wild herbivores are in decline due to poaching and land‐use change while livestock numbers are increasing. These changes in density and composition alter savanna vegetation. There are likely indirect cascading effects of altered vegetation on savanna arthropods, but our understanding is limited despite their pivotal role in ecosystem functioning.
2. We evaluate how differences in mammalian herbivory affect terrestrial arthropods in a semiarid Kenyan savanna. We sampled ground‐active arthropods (focusing on ants) in six herbivory treatments ranging from high‐intensity herbivory to complete exclusion of large herbivores.
3. Ant abundance and richness were not affected by herbivory treatments, but the community composition of ants and arthropods differed at extremely high and low levels of herbivory due to indirect impacts on vegetation.
4. Community composition changes occurred under extremely high levels of herbivory because the resulting short‐grass communities and patches of bare ground led to high species turnover in ants. By contrast, extremely low herbivory promoted woody encroachment that led to the loss of savanna specialists via both species turnover and nestedness.
5. We conclude that cascading effects of mammalian herbivory play only a relatively small role in shaping savanna arthropod communities, except at extreme levels of herbivory. However, the occurrence of savannas with these extreme levels of herbivory, both high and low, is likely to increase in the future, which may lead to more widespread changes in ecosystem functioning as a consequence of shifts in arthropod community composition.

## Graphical abstract

This study investigates how large mammalian herbivores shape arthropod communities in African savannas, using a broad gradient of herbivory types and intensities to assess these effects under real‐world, non‐experimental conditions.

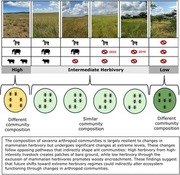

## 1 INTRODUCTION

Large mammalian herbivores have unique ecological roles across tropical ecosystems by exercising top‐down effects on plant biomass, structure and composition at levels that cannot be achieved by small vertebrate or arthropod herbivores (Forbes et al., 2019; Pringle et al., 2023). These impacts on vegetation vary across mammalian herbivores depending on factors such as herbivore body size and feeding type, that is, grazing, browsing or mixed‐feeding (Staver et al., 2021; Venter et al., 2019). Yet, many large herbivores are declining in abundance and are threatened by local extinction, with many regions losing their native herbivores due to anthropogenic environmental changes (Malhi et al., 2016; Ripple et al., 2015), for example, protected areas in Sub‐Saharan Africa have experienced significant declines in large herbivore populations (Craigie et al., 2010) while mammalian herbivores have similarly declined across South and Southeast Asia (Sankaran & Ahrestani, 2016). These declines in large herbivores can affect ecosystem structure and functioning as demonstrated by exclosure experiments conducted globally (Forbes et al., 2019). Simultaneously, the abundance of domesticated large herbivores (i.e. livestock) is increasing, often replacing or competing with native herbivores (Ripple et al., 2015). However, herbivory by livestock differs from native large herbivores due to differences in feeding types and body sizes, for example, there is no livestock equivalent to elephants (Pringle et al., 2023). This shift in herbivory affects all terrestrial biomes, but is likely to have especially strong effects on savanna biomes where large mammalian herbivory plays an important ecosystem role (Malhi et al., 2016).

Savannas, characterized by a continuous C4‐grass cover, shade‐intolerant plant species and highly variable woody cover (Bond, 2021; Parr et al., 2014), have evolved in response to the presence of large herbivores and fire (Bond, 2019), which maintain their character as open ecosystems by suppressing forest seedlings or reducing tree cover through megaherbivores (Staver et al., 2021). While African savannas are among the few places that remain with a diverse and abundant megafauna, including extant megaherbivores (Malhi et al., 2016; Ripple et al., 2015), native herbivore populations are declining due to anthropogenic factors, principally poaching and habitat loss (Smith et al., 2020; Wiethase et al., 2023). Partial or complete exclusion of large herbivores promotes woody thickening (Devine et al., 2017; Stevens et al., 2017; Venter et al., 2018), with increasing tree cover resulting in shading of the ground layer and the subsequent loss of the herbaceous layer and light‐loving plant taxa (Bond, 2019, 2021). Livestock can partly replace the ecological roles of native herbivores but can also lead to less structurally complex habitats which are dominated by short‐grass communities with low forage value and greater bare ground (Veblen et al., 2016; Voysey et al., 2024; Wiethase et al., 2023). While the patterns of native herbivores and livestock on savanna vegetation are generally understood, much less is known about the indirect effects on other taxa.

Large herbivores can directly impact other taxa (e.g. ingestion of insects on foliage), but most effects are indirect through changes in vegetation composition and structure (Foster et al., 2014; van Klink et al., 2015). These effects vary across taxa; for example, bird species richness in savannas can increase with increasing woody cover following large herbivore exclusion, as certain species benefit from greater habitat complexity (Ogada et al., 2008), while many other savanna taxa are shade‐intolerant and respond negatively to increased woody cover (Smit & Prins, 2015). Among the taxa influenced by herbivore‐driven vegetation changes, terrestrial arthropods play particularly critical roles in savannas. The indirect effects of mammalian herbivory alter arthropod abundance, species richness and community composition, including beetles, spiders and grasshoppers (Goheen et al., 2018; Prendini et al., 1996; Pryke et al., 2016). Terrestrial arthropods are not only key herbivores themselves but also play critical roles as ecosystem engineers, shaping habitats and influencing nutrient cycling (Andersen et al., 2003; Parr et al., 2016) and changes in their community composition can have especially significant implications for ecosystem functioning. Even when species richness remains constant, shifts in the relative abundances or identities of taxa can alter key ecosystem processes (McCary et al., 2021; Spaak et al., 2017). Better understanding of how large herbivores affect savanna arthropods is therefore essential, as their responses may have far‐reaching consequences for ecosystem functioning. However, despite numerous studies focusing on few arthropod taxa such as beetles (Pryke et al., 2016, 2022) or spiders (Warui et al., 2005), there is a critical lack of empirical evidence for arthropod responses to altered and contrasting herbivory. Comprehensive assessments of entire arthropod communities, especially those that consider a full spectrum of different types of large mammalian herbivory, remain limited but are crucial to understand the potential consequences of herbivory shifts for ecosystem function.

Ants (Hymenoptera: Formicidae) have been widely used to assess changes in savanna ecosystems, and research in savannas and rangelands suggests that ants show consistent responses to grazing (Hoffmann, 2010; Hoffmann & James, 2011): soil and vegetation type are thought to influence ant community composition more than grazing, and grazing alters community structure but not necessarily species richness or abundance. However, most studies have been conducted in Australian savannas (Arcoverde et al., 2017; Hoffmann, 2000; Hoffmann & James, 2011) where ant communities are generally resilient to different intensities of livestock grazing (but see Hoffmann, 2000), although relative to other arthropod taxa ants are considered more responsive to grazing treatments (Neilly et al., 2020). These conclusions are strongly based on the impact of livestock rather than native herbivores, and Australian savannas lack the extant megafauna of African savannas, and consequently, mammalian herbivory may play less of a role in Australian ecosystems than in Africa. Studies on the relationship between native herbivores and ant diversity from African savannas are rare, but indicate that high‐intensity grazing leads to substantial changes in ant diversity and ecological functions (Purdon et al., 2019). There is evidence also that woody encroachment, a consequence of exclusion of herbivores, promotes distinctly different ant communities (Parr et al., 2012). While herbivory is known to shape communities of certain arthropod taxa, the effects on whole arthropod communities in general and on ants in particular remain poorly understood in the context of African savannas, limiting our ability to predict the ecological consequences of the ongoing shift in mammalian herbivory.

In this study, we investigate how savanna arthropods are affected by variation in large mammalian herbivore type and herbivory pressure, with a particular focus on ants. Ants were selected for detailed analysis due to their relative ease of identification to lower taxonomic levels, their varied roles in the ecosystem and the existence of clear predictions regarding their sensitivity to herbivory. We test the predictions that (1) ant abundance and richness will vary little in relation to large mammalian herbivory, in accordance with observations from other savannas, but that (2) ant and arthropod community composition will change, driven by herbivore‐induced alteration of vegetation and microhabitat and that (3) changes in ant and arthropod community composition will be explained by measurable vegetation changes in response to herbivory.

We sample arthropods in a semi‐arid savanna landscape that is subject to different management and land‐use types and has well‐defined areas that allow either access of livestock at different intensities, native herbivores or partial or full exclusion of large herbivores, allowing us to examine a wide range of variation in mammalian herbivory typical of African savannas.

## 2 METHODS

### 2.1 Study site and herbivory treatments

**FIGURE 1:**
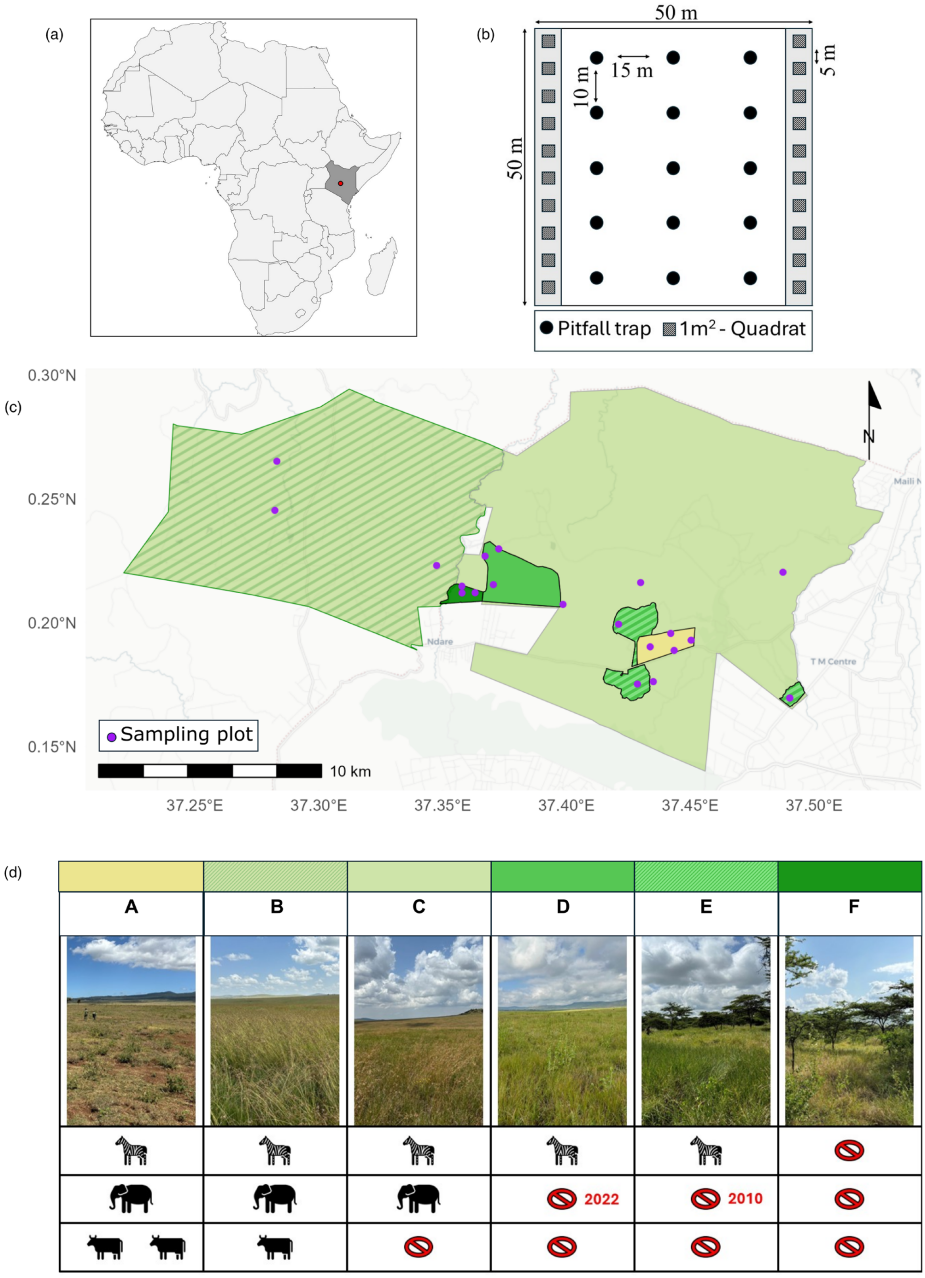
(a) Location of our study landscape in Kenya; (b) Schematic of our sampling design for arthropods (pitfall traps) and vegetation (quadrats) in each plot; (c) Map of study area showing different herbivory treatments across the study landscape and the location of the sampling plots (pink dots); (d) Schematic figure of the six herbivory treatments A–F (see Table 1): Zebra symbols represent herbivory by native mesoherbivores, elephant symbols represent herbivory by native megaherbivores and cattle symbols represent herbivory by domestic livestock. Red prohibition signs represent exclusion of these large mammal groups.

This study was conducted at Lewa Wildlife Conservancy (0°13′31″ N, 37°26′27″ E) and Borana Conservancy (0°13′24″ N, 37°19′07″ E) in the Meru and Laikipia counties in Kenya (Figure 1a,c). This savanna rangeland landscape north of Mount Kenya has an elevation spanning 1400–2200 m a.s.l. and average annual rainfall between 400 and 600 mm, with pronounced wet seasons peaking in April and November (average monthly rainfall of 100 mm) and a dry season between July and August (average monthly rainfall >10 mm) (Giesen et al., 2017). The average temperature ranges from 12°C/24°C (min/max) in the dry season to 13°C/26°C in the wet season. Although fire might have played a larger role in the past, fires are suppressed by the Conservancy managements and herbivore pressure through large mammals is considered more important in determining woody cover in this landscape (Giesen et al., 2017). Dominant tree species are *Vachellia drepanolobium*, *Vachellia mellifera* and *Vachellia seyal* (Dinerstein et al., 2017). Soil types can be categorized broadly into red sandy soils and black cotton soils. All sampling sites were located on black cotton soil (clay: 43.9%–66.8%, silt: 20%–30.7%, sand: 13.1%–27.8%). All fieldwork was conducted under Government of Kenya NACOSTI permit (NACOSTI/P/25/4175330). Ethical approval for this study was granted by the Chuka University Institutional Ethics Review Committee (NACOSTI/NBC/AC‐0812).

We surveyed ground‐dwelling arthropods in six treatments that differ in herbivory type and intensity (Table 1). We studied these six treatments because they span a gradient from extremely high to extremely low levels of herbivory and represent variation in herbivory present in African savannas, including livestock (consisting of cattle, goats, sheep and camels).

**TABLE 1:** Description of the six herbivory treatments.

Treatment type	Treatment description and number of sampling plots per treatment	Size of treatment
(A) Wildlife and high intensity livestock	Continuous area with unmanaged intensive livestock with near permanent presence of goats and sheep, occasional presence of cattle and camels and presence of unrestricted native wildlife. Number of sampling plots: 4	261 ha
(B) Wildlife and livestock	Continuous area with managed and regulated livestock consisting of rotational cattle grazing and unrestricted native wildlife. Rotational grazing is a strategy of time‐controlled rotations of high‐density groups of cattle, used to lessen livestock impacts by reducing grazing selectivity and avoid overgrazing by frequent rotations (Crawford et al., 2019). Number of sampling plots: 3	11,800 ha
(C) Wildlife	Continuous area with unrestricted native wildlife, but no livestock allowed. Number of sampling plots: 4	19,709 ha
(D) Wildlife—No megaherbivores (young)	Continuous area with native wildlife. Megaherbivores are excluded by elephant‐exclusion fences (established 2022). Number of sampling plots: 3	684 ha
(E) Wildlife—No megaherbivores (old)	Three separate areas with native wildlife. Megaherbivores are excluded by elephant‐exclusion fences (established between 2008 and 2010). Number of sampling plots: 3	532 ha (65 ha + 211 ha + 256 ha)
(F) Full exclosure	Continuous area with full exclusion fences for large mammalian herbivores (established 1970 and re‐fenced in 2023). Number of sampling plots: 3	109 ha

We surveyed arthropods in three 50 × 50 m plots per treatment (Figure 1c), except at treatments A and C, where we sampled four plots (i.e. 20 plots in total). The treatments and plots occurred across the study area, with a minimum distance of 300 m between plots. Importantly, our scenarios of herbivore type and intensity reflect real‐world examples, but this reality necessarily means we have a limited sample size for some treatments. See Table S1 for a more detailed description of these six herbivory treatments and Table S2 for a full list of native mammalian wildlife present in the study landscape.

### 2.2 Arthropod sampling

At each of the 20 plots, we sampled ground active arthropods with pitfall traps between June and August 2024 (dry season when sites are accessible). Each 50 × 50 m plot had 15 pitfall traps set out in a grid of three parallel transects each with five traps 10 m apart and 15 m between transects (Figure 1b). The pitfall traps were 300 mL plastic containers (80 mm diameter), part‐filled with a 50% solution of propylene glycol (Parr et al., 2004) and a drop of detergent to reduce surface tension (Netshilaphala et al., 2005). A plastic plate was fixed with a steel wire circa 10 cm above each trap as a rain cover. Traps were deployed for 3 days, and then, the collected arthropods were transferred into 70% ethanol. Arthropods were subsequently sorted under a stereomicroscope and identified to order level using ‘*Field Guide to Insects of South Africa*’ (Picker, 2012), with ants (Hymenoptera: Formicidae) identified to genus level using *Ants of Africa and Madagascar: A Guide to the Genera* (Fisher & Bolton, 2016) and subsequently assigned to (morpho)species. A reference collection is deposited at the University of Liverpool, United Kingdom.

### 2.3 Measuring local vegetation

We surveyed ground vegetation in each plot and recorded five variables: percentage cover of bare ground (estimated by eye), grass and forb diversity (number of species present), height of herbaceous vegetation (cm) and tree density (ha). In each plot, we surveyed the first three variables along two 50‐m transects running parallel to each other on opposite edges of the plot (Figure 1b). These three vegetation variables were measured in a 1‐m^2^ quadrat placed every 5 m along the two transects, that is, 20 vegetation measurements per variable per 50 × 50 m plot. We used the mean of these 20 measurements of bare ground cover for analysis and the occurrence of all grass and forb species across the 20 measurements to calculate plot‐level species richness. We measured herbaceous vegetation height using a Disc Pasture Meter (Trollope & Potgieter, 1986) with a disc diameter of 45.5 cm on 100 points equally distributed across each plot and used the mean of these 100 measurements for analysis. All trees in each 50 × 50 m plot with a diameter of 5 cm or greater (measured at a height of 50 cm from the ground, as many trees in these systems are multistemmed above this point) were recorded to calculate tree density (trees ha^−1^).

### 2.4 Statistical analysis

Our statistical analyses of richness and abundance (prediction 1) focused on ants to test if predictions from other savanna systems are applicable to savannas shaped by mammalian herbivory. For the analyses of community composition (predictions 2 and 3) where we expected more nuanced patterns in response to herbivory and vegetation parameters, we included arthropods to confirm if patterns observed for ant communities were robust enough to also be detected on a taxonomically much coarser level. We assessed sampling completeness of ants by constructing species accumulation curves per treatment using sample size‐based rarefaction and extrapolation with the R package ‘iNEXT’ (Chao et al., 2016), with pitfall traps representing samples (more details in Figure S1). For the remaining analysis, data on ant species and arthropod order counts were each pooled together from all individual pitfall traps for each plot (3–4 plots per treatment, 20 plots in total).

To test the influence of herbivory on ant abundance and species richness, we used one‐way analysis of variance (ANOVA) to test for differences among our six herbivory treatments. Abundance and richness were natural log‐transformed prior to analysis to meet assumptions of normality. If results were significant, we used Tukey's honest significant differences to test for pairwise differences between treatments.

We explored how ant and arthropod community composition varied among our six treatments by visualizing variation in the composition, using non‐metric multidimensional scaling (NMDS) and the Bray–Curtis dissimilarity index (separate analyses for arthropods and ants). We used a fourth‐root transformation of the ant data to reduce the influence of highly abundant ant species and allow greater contribution from rare species (Clarke, 1993). To test for significant clustering in community composition among herbivory treatments, we used a Permutation Multivariate Analysis of Variance, computing 9999 permutations. Where differences in species community composition among herbivory treatments were found, we tested whether differences were due to turnover (replacement of species between treatments) or nestedness (species loss between treatments). Since we found differences in community composition only at the extreme levels of our herbivory treatments, we combined our six treatments into three herbivory categories: high (treatment A), intermediate (treatments B–E) and low (treatment F). To account for unequal sampling effort, we applied a resampling approach, randomly selecting for each pairwise comparison the smallest number of plots shared among the compared categories; this was repeated 1000 times to estimate mean values and 95% confidence intervals. For each iteration, we constructed an aggregated presence–absence species matrix by combining the data from the selected plots. Following Baselga (2010), we used the *betapart* R package (Baselga & Orme, 2012) to compute pairwise beta diversity partitioning based on the Jaccard index to calculate the turnover (*β*_JTU_), nestedness (*β*_JNE_) and total beta diversity (*β*_JAC_) between the three herbivory categories.

To investigate whether differences in vegetation explain variation in arthropod community patterns among the six herbivory treatments (prediction 3), we examined how our five vegetation variables differed among treatments, using one‐way ANOVA or Kruskal–Wallis tests. If results were significant, we conducted Tukey's post hoc test or Dunn's post hoc test to identify pairwise differences between treatments. These analyses of vegetation were based on three plots per treatment as vegetation data were not available for two sampling plots. We used canonical correspondence analysis (CCA) to assess relationships between community composition and vegetation variables (separate analyses for ants and arthropods). As recommended to avoid overfitting (Braak, 1986), we used principal component analysis (PCA) to reduce the five vegetation variables to PCA factors. Variables were standardized (*z*‐scores), and multicollinearity was assessed using variance inflation factors (VIFs; all VIFs <5). The first two PCA axes (which together explained 66.8% of the cumulative variance) served as constraints in the CCA. Ant and arthropod data were Hellinger‐transformed to reduce the influence of dominant species and orders (Legendre & Gallagher, 2001). The significance of CCA axes in explaining community composition among treatments was tested with 9999 Monte Carlo permutations.

All statistical analyses were performed using R version 4.4.3 (R Core Team, 2025).

## 3 RESULTS

We surveyed 20 plots across six treatments that varied in large mammalian herbivory, and we collected a total of 9878 ants, belonging to 92 (morpho)species and 27 genera, and 7234 other arthropods belonging to 22 Orders. The most common ant genera were *Pheidole* (79% of all individuals), *Monomorium* (5%) and *Tetramorium* (4.6%), and the most species‐rich genera were *Pheidole* (14 species), *Tetramorium* (14), *Camponotus* (11) and *Monomorium* (10). The most common arthropods were Collembola (2286 individuals), Hemiptera (1092), Diptera (1040) and Coleoptera (847; Table S3). Species accumulation curves for ants approached asymptotic plateaus for all treatments, indicating sufficient sampling completeness for all treatments (Figure S1).

### 3.1 Effect of herbivory on ant richness and abundance

**FIGURE 2:**
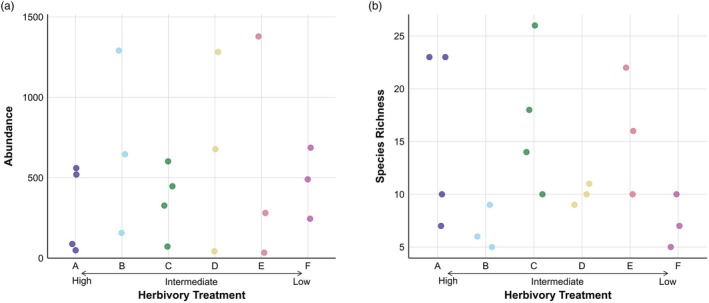
(a) Ant abundance and (b) species richness in the six herbivory treatments (A–F, see Table 1 for descriptions of treatments). Each data point represents a plot.

We found no significant differences in ant abundance among herbivory treatments (ANOVA abundance; *F*_5,14_ = 0.278, *p* = 0.918) but considerable variation among plots within treatments (Figure 2a); for example, plots with the highest (1379 individuals) and lowest abundance (34) occurred in the same treatment (E). Differences in abundance were driven by changes in highly abundant *Pheidole* species. In contrast to abundance, species richness differed significantly among treatments (*F*_5,14_ = 3.079, *p* = 0.044) but not in respect to the herbivory gradient, with treatments A, C and E having higher species richness than B, D and F, but no significant differences between pairs of treatments (Figure 2b; for a more detailed overview of abundance and species richness per plot and post hoc comparisons for species richness, see Tables S4 and S5). Species richness varied considerably among plots within treatments, in particular for treatment A, where the number of species at plot level was either high or low.

### 3.2 Effect of herbivory on community composition

**FIGURE 3:**
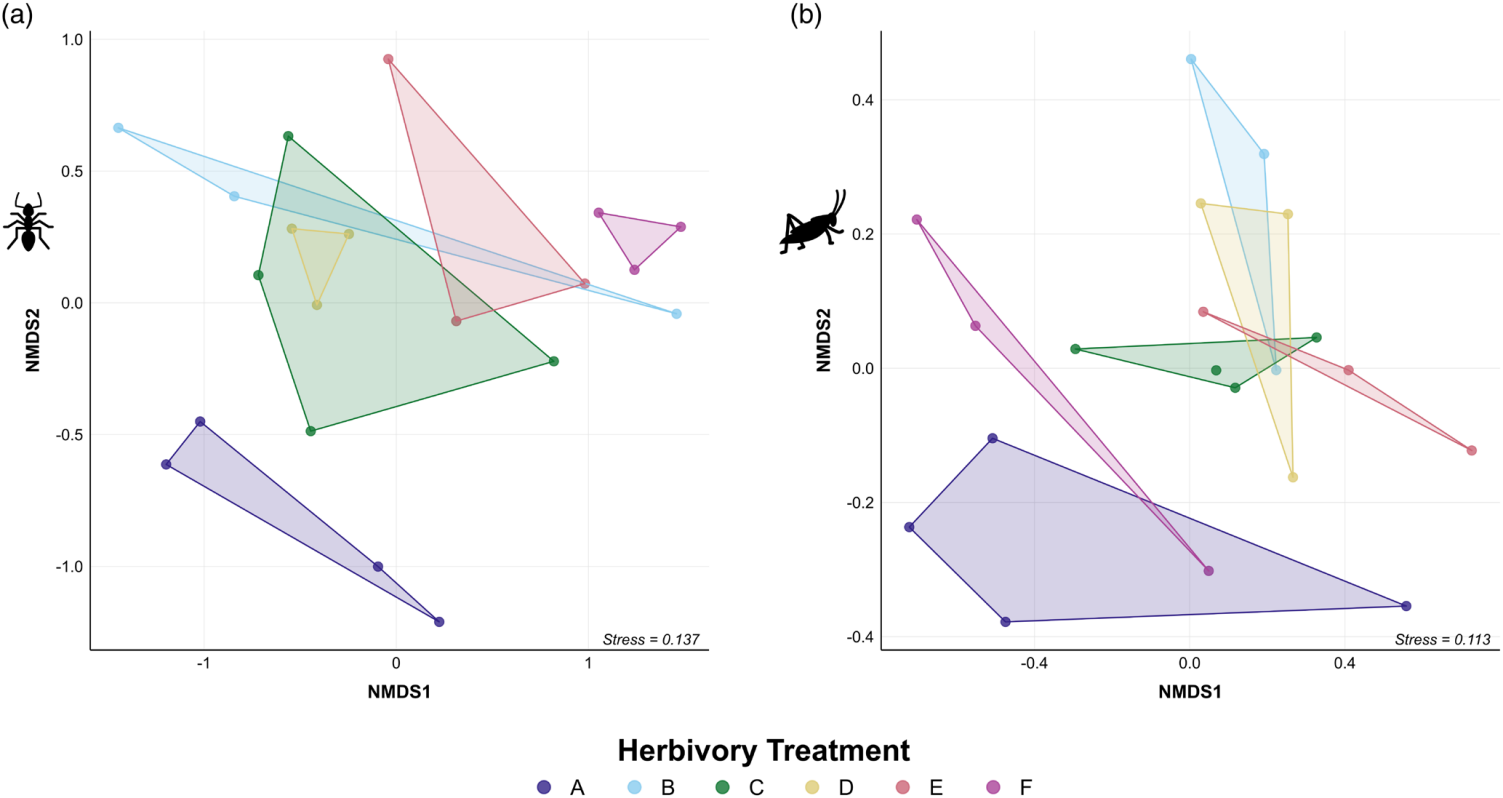
Non‐metric multidimensional scaling (NMDS) ordinations for (a) ant composition and (b) terrestrial arthropod order composition across the six herbivory treatments (A–F, see Table 1 for descriptions of treatments) based on Bray–Curtis index. Each point represents a plot. The axes represent ordination dimensions and relative dissimilarities. Points that cluster closely together indicate similar communities. The stress values of 0.137 and 0.113 indicate a good representation of the community structures.

Ant and arthropod community composition differed among treatments (PERMOVA ants: *F* = 2.00, *R* ^2^ = 0.42, *p* = 0.002; arthropods: *F* = 2.55, *R* ^2^ = 0.48, *p* = 0.009), with the NMDS (ants, stress: 0.137; arthropods, stress: 0.113) revealing that plots at extreme levels of herbivory (i.e. treatments A and F) clustered separately from the other four treatments which clustered together (Figure 3a,b). For ants, treatments A and F are positioned at opposite ends of the ordination, indicating that these plots not only differed from the intermediate treatments but also from each other. However, for arthropods, extreme treatments A and F clustered in closer proximity to each other, suggesting extreme levels of herbivory produce more similar arthropod communities regardless of the type of extreme herbivory (high or low).

Beta division partitioning of the ant communities indicated that changes between high (treatment A) and intermediate (treatments B–E) herbivory categories were due to species turnover, indicating that high intensity herbivory promotes savanna ant species that are not found at other treatments. In contrast, ant community changes between intermediate and low (treatment F) herbivory categories were due to turnover, but with a greater role of nestedness, indicating that low levels of herbivory result in the loss of savanna ant species as well as promoting species not found in other treatments (Table 2). For arthropods, the communities in high and low herbivory categories changed in more similar ways in relation to the intermediate category, suggesting that communities in extreme herbivory treatments are due to both replacement and the loss of arthropod orders. We conclude that intermediate levels of herbivory have little effect on ant and arthropod community composition, but that extremely high and low levels of herbivory lead to communities distinctly different from those found at intermediate levels.

**TABLE 2:** Pairwise comparison of Jaccard dissimilarity between the three herbivory categories (high [treatment A], intermediate [B–E], low [F]) for ants and arthropods. Values represent mean Jaccard dissimilarity partitioned into turnover and nestedness components, with 95% confidence intervals shown in brackets.

	Herbivory comparison	Beta diversity (*β*_JAC_)	Turnover (*β*_JTU_)	Nestedness (*β*_JNE_)
Ants	High vs. intermediate	0.755 (0.652–0.850)	0.724 (0.623–0.810)	0.030 (0.002–0.090)
	Low vs. intermediate	0.850 (0.763–0.940)	0.757 (0.545–0.933)	0.093 (0.004–0.228)
Arthropods	High vs. intermediate	0.365 (0.263–0.476)	0.293 (0.125–0.421)	0.072 (0–0.175)
	Low vs. intermediate	0.347 (0.235–0.500)	0.277 (0.133–0.444)	0.070 (0–0.179)

### 3.3 Effects of herbivory‐driven vegetation changes on arthropod communities

**FIGURE 4:**
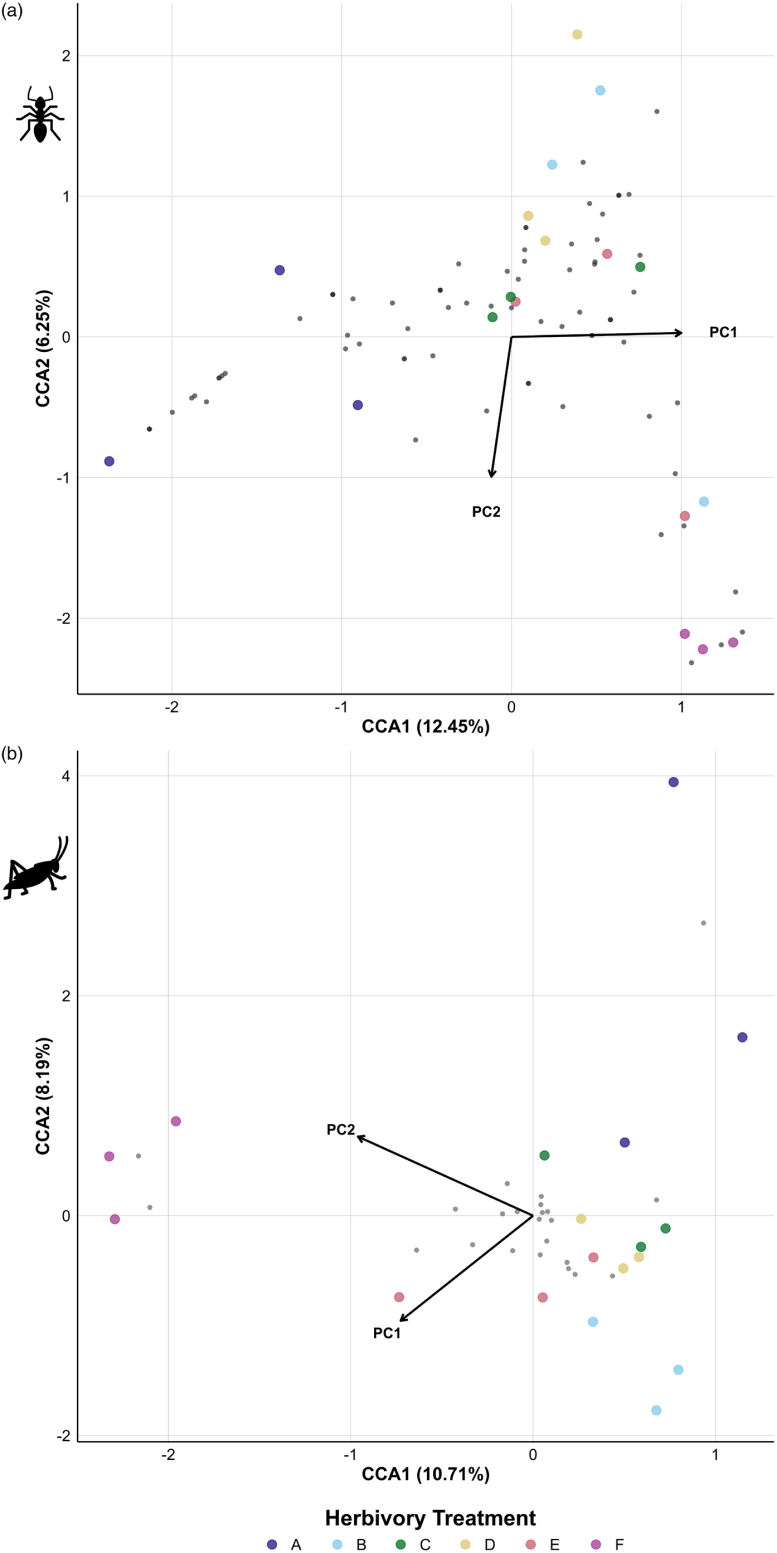
Canonical correspondence analysis to explore (a) ant species and (b) arthropod orders in six herbivory treatments in relation to vegetation factors (PC1 and PC2, see Table 3 for factor loadings). Grey dots represent (a) ant species and (b) arthropod orders. Coloured dots represent plots in the six herbivory treatments A–F (see Table 1 for treatment descriptions).

Herbivory altered vegetation structure and composition, with higher herbivory leading to shorter herbaceous layer (*F* = 4.51, *R* ^2^ = 0.65, *p =* 0.015), increased grass richness (*F* = 5.68, *R* ^2^ = 0.70, *p* = 0.006) and lower tree density (*χ* ^2^ = 14.9, df = 5, *p =* 0.011). Differences in bare soil (*F* = 4.81, *R* ^2^ = 0.67, *p =* 0.012) occurred between treatments but not in respect to the herbivory gradient, with Treatments A and C having high and Treatment B having low cover of bare soil, while forb richness was not significantly altered by herbivory (*F* = 2.51, *R* ^2^ = 0.51, *p* = 0.089; Figures S2–S6; Tables S6 and S7). The PCA revealed that PCA factors 1 and 2 explained 36.04% and 29.89% of the total variance, respectively, with PC1 reflecting grass diversity and herbaceous cover (lower values represent lower grass richness with less bare ground but high herbaceous vegetation height) and PC2 increasing with increasing woody cover (higher tree density and forb richness with low herbaceous vegetation height; Table 3; Figure S7). Ant community composition was associated with these vegetation factors (canonical correspondence analysis [CCA], Monte Carlo permutation test, *p* = 0.003), especially grass diversity and cover (*p* = 0.0002), but vegetation factors explained only 12.45% (CCA1) to 6.25% (CCA2) of total variance, indicating that vegetation factors play only a small role in structuring ant communities (Figure 4a). Similarly, arthropod community composition was linked to vegetation factors (Monte Carlo permutation test, *p* = 0.013) but these also explained only 10.71% (CCA1) to 8.19% (CCA2) of the variance, suggesting a similarly minor influence of vegetation on community structure (Figure 4b).

**TABLE 3:** Loadings of five vegetation variables on the first two principal components, which represent gradients in grass diversity and cover (PC1) and woody cover (PC2).

Variable	PC1 (grass diversity and cover)	PC2 (woody cover)
Grass richness	−0.551	−0.177
Forb richness	0.153	0.526
Herbaceous vegetation height	0.447	−0.419
Bare ground	−0.596	−0.346
Tree density	0.342	0.630

## 4 DISCUSSION

Large mammalian herbivores are key consumers in African savannas, shaping vegetation structure and diversity (Bond, 2019), but anthropogenic changes are driving shifts from native herbivores to more intensive livestock herbivory or to complete herbivore exclusion/loss (Staver et al., 2021), with indirect effects for other savanna taxa including arthropods (Goheen et al., 2018). Our study shows that arthropods in African savannas are generally resistant to moderate changes in large herbivore type but are affected by extreme levels of herbivory (very high levels of herbivory or none). Our findings support our initial predictions based on findings from other geographic regions and show that herbivore impacts on ants primarily manifest through shifts in community composition rather than through changes in overall abundance or species richness. These shifts in ant and arthropod community composition did not follow any gradient of herbivory intensity or composition of herbivores but only occurred at the most extreme levels, and importantly, livestock did not necessarily have a detrimental effect on arthropod taxa. We confirmed our prediction that vegetation parameters drive these shifts in arthropod composition, which are due to changes in grass richness and cover under extremely high herbivory and increased woody cover under extremely low herbivory. The measured parameters only explain a small part of the variation in community structure, however, likely because the intermediate levels of herbivory had similar effects on the measured vegetation parameters. While the extreme treatments are characterized by lower grass richness and higher tree density (F) or a lower height of the herbaceous layer (A), the intermediate treatments do not differ across these parameters. In the case of the livestock grazing (B), this could indicate that the rotational grazing technique used reduces the livestock impact, and therefore, the density of livestock in this system is not having a measurable effect on the vegetation. The treatments where megaherbivores are excluded (D and E) were expected to primarily change through an initial increase in tree density, which subsequently negatively affects the shade‐intolerant grasses. The significant increase in trees on the older treatment (E, 14–16 years of megaherbivore suppression) shows this process, but the lower number of trees and the high number of grass species compared with treatment F suggest that the effects of woody encroachment are more apparent after a longer period of time. Treatment D shows that a short‐term (2 years) exclusion of megaherbivores has no discernible effect on vegetation.

We find that extreme levels of herbivory, both high (treatment A) and low (F), produce ant communities that are distinct from intermediate levels of herbivory as well as from each other. These findings are due to the effects of herbivory on vegetation and habitat openness (i.e. tree density). High herbivory promotes less structurally complex habitats by creating areas of short grass with bare ground, while suppression of herbivory creates more structurally complex habitats by promoting trees (Bond, 2019; Hempson et al., 2015); importantly, these differences in habitat complexity and openness are key determinants of ant communities (Andersen, 2019). Our findings support previous studies which show that high‐intensity grazing (e.g. grazing‐lawns) and woody encroachment promote different ant communities compared with open savanna habitats (Parr et al., 2012; Purdon et al., 2019). It is striking that we found differences in the arthropod communities between the intermediate herbivory treatments and the extremes given the coarse level of taxonomic identification (order level); but this emphasizes the resilience of arthropods to intermediate levels of herbivory (Figure S8a,b). For the arthropod communities, it is possible that vegetation parameters such as grass height or biomass play more important roles than habitat openness and that taxa such as Orthoptera (Figure S9) are filtered out at both extreme ends of herbivory where grass height or biomass is likely to be reduced by high intensity herbivory and woody encroachment (Prendini et al., 1996; van Klink et al., 2015). Other taxa may benefit from these changes, resulting in arthropod communities containing species not found in open savanna habitats.

From a functional perspective, an important ecological question is how extreme human‐induced herbivory alters ant communities relative to the more stable communities observed at intermediate levels of herbivory. Our study shows that the difference between high‐intensity and intermediate treatments is mainly driven by species replacement, including species belonging to open‐habitat‐associated savanna ant genera such as *Messor*, *Meranoplus* and *Ocymyrmex*, which provide important ecosystem services such as seed dispersal (Bolton, 1982; Plowes et al., 2013), being promoted under high‐intensity herbivory. The differences in ant communities between herbivore exclusion and intermediate treatments are mainly driven by species replacement, but also to some extent by loss of savanna species due to woody encroachment. However, despite the high tree density in the full exclosure treatment (F), we did not find that these plots were colonized by ant genera associated with forest habitats such as *Strumigenys* (leaf‐litter dwelling) or *Cataulacus*. In case of our study, the relatively close proximity of true forest (<5 km) and age of the exclosures means that the lack of true forest ant species is unlikely to be due to dispersal limitations. Our study suggests, therefore, that full suppression of large mammalian herbivory (or defaunation) can result in an impoverished ant community dominated by savanna species that can tolerate more closed habitats rather than by promoting forest‐adapted ant communities.

Our study was designed in a landscape with established ‘real‐life’ variation in mammalian herbivory, as opposed to, for example, small‐scale experimental treatments, and so, we faced a number of challenges, including limited replication of treatments we could sample, variation in age structure of herbivore exclosures and lack of detailed quantitative information about livestock populations and densities. However, despite the limited statistical power of our study from surveys of 20 plots, the insights gained are valuable considering that African savannas cover >50% of the continent and are experiencing widespread and dramatic changes in large mammalian herbivore populations (Hempson et al., 2017); we hope our findings will encourage further studies. Due to logistical limitations, our sampling was conducted at the end of the wet and the beginning of the dry season, although typically arthropod activity peaks in the wet season. It is, therefore, possible that there may be a more pronounced difference in arthropod and ant communities among the intermediate herbivory categories during the period of peak arthropod activity.

Caution should also be exercised when applying our findings from a semi‐arid savanna to other savanna types, because herbivory impacts are influenced by rainfall, soil and fire regimes (Archibald & Hempson, 2016). For example, in mesic savannas (>750 mm rainfall), fire is the key consumer of vegetation, meaning it has a stronger influence on plant community structure than herbivory (Bond, 2019). Consequently, herbivore exclusion may have weaker impacts in these ecosystems, while high‐intensity herbivory may have even stronger indirect effects on arthropods. We conducted our study in a landscape with productive clay‐rich black cotton soils which mitigate herbivore impacts on vegetation, and the indirect effects on other taxa, compared with less nutrient‐rich soils with a high sand content (i.e. ‘red‐sandy soils’) which are likely to take significantly longer to recover after intense periods of herbivory (Goheen et al., 2018) and therefore will be even more affected by high‐intensity herbivory. Furthermore, our sampling was conducted 1 year after the end of a severe drought period from 2020 to 2023 (Asokan et al., 2025), which can reduce livestock and wildlife densities through increased mortality rates or migration to more resource‐rich areas (Augustine, 2010; Bogale & Erena, 2022), and exacerbate the effects of overgrazing (Smit et al., 2025). However, given that our sampling sites are located on productive black cotton soils, most treatments have been established for many years and droughts are frequent in these rangelands, we do not assume that the recent drought had a fundamental effect on differences among treatments.

Our findings have several implications for conservation and management in semi‐arid savannas. Modern African landscapes represent a full spectrum ranging from livestock‐only systems, to native wildlife‐only systems, to systems lacking all large mammalian herbivores. It is important to highlight that our study system did not include livestock‐only systems, and our treatments with livestock always included native wildlife. A key implication for conservation from our study is that maintaining at least some level of mammalian herbivory is important to prevent woody encroachment and the associated changes in ant diversity. This is particularly important as woody encroachment is predicted to increase in the future due to the ongoing loss of native mammalian herbivores, but also due to other drivers such as fire suppression and rising CO_2_ (Stevens et al., 2016, 2017). We also find that supplementing native wildlife with livestock is not necessarily detrimental for arthropod diversity, which was resilient to intermediate levels of herbivory, and that high intensity livestock (i.e. treatment A) can promote ant diversity within landscapes by supporting savanna specialist species. Heavily grazed areas with extensive patches of bare ground are often considered ecologically compromised or degraded, but our study shows that having at least some of these areas within the landscape is important from an arthropod biodiversity perspective. Taken together, our findings suggest that, for some taxonomic groups such as ants, there is not necessarily a conflict between pastoralism and biodiversity conservation in these rangelands, which is especially important given that these rangelands are widely used by local communities for livestock grazing.

High‐intensity grazing via livestock is likely to expand across African savannas due to a growing human population and increasing demand for livestock products, but our findings suggest that this may not necessarily have detrimental effects on arthropod diversity, provided there is variation in herbivory levels at landscape scales. However, the shifts we observed in ant and arthropod community composition imply potential changes in ecosystem functions, which require further investigation. The persistence of native herbivores, as well as livestock, may be vital for maintaining ecosystem functions in these increasingly managed landscapes, and so we must remain vigilant to the long‐term consequences of losing native mammalian herbivores.

## AUTHOR CONTRIBUTIONS

**Bjoern Erik Matthies**: Conceptualization, methodology, investigation, formal analysis, writing—original draft. **Nicola Stevens**: Conceptualization and methodology. **Jane K. Hill**: Conceptualization, writing—review and editing and supervision. **Bosco Leturuka**: Investigation and project administration. **Margaret Njuguna**: Investigation and project administration. **Lucy K. Smyth**: Data curation and project administration. **Matthew S. Rogan**: Conceptualization and data curation. **Catherine W. Machungo**: Methodology and project administration. **Jonathan E. M. Baillie**: Conceptualization, resources and project administration. **Jafford N. Rithaa**: Supervision and project administration. **Catherine L. Parr**: Conceptualization, methodology, writing—review and editing and supervision.

## CONFLICT OF INTEREST STATEMENT

The authors have no conflict of interest to declare.

## STATEMENT ON INCLUSION

Our study brings together authors from several different countries, including scientists based in the country where the study was carried out. All authors were engaged early on with the research and study design to ensure that the diverse sets of perspectives they represent were considered from the onset. Whenever relevant, literature published by scientists from the region was cited; efforts were made to consider relevant work published in the local language.

## Supporting information


**Table S1.** Description of the study landscape and distribution of large mammalian herbivory treatments.
**Table S2.** Numbers of large mammalian herbivores counted in Lewa and Borana 2022–2024 (Game Count Report Lewa Borana 2024).
**Table S3.** List of invertebrate orders found across the 20 sampling plots, with total abundance across all plots.
**Table S4.** Ant abundance and species richness for each of the 20 sampling plots across six treatments (A–F, see Table 1 for descriptions of treatments).
**Table S5.** Tukey HSD post‐hoc pair‐wise comparisons between six treatments (A–F) for ant species richness (natural‐log‐transformed).
**Table S6.** Tukey HSD post‐hoc pair‐wise comparisons between six treatments (A–F) for the environmental variables: mean height of herbaceous layer, grass richness (natural‐log‐transformed) and mean bare soil (natural‐log‐transformed).
**Table S7.** Dunn's test pair‐wise comparisons between six treatments (A–F) for tree density using Benjamini–Hochberg correction.
**Figure S1.** Species accumulation curves for the six herbivory treatments (A–F).
**Figure S2.** Scatter plot of mean height (cm) of herbaceous vegetation in the six herbivory treatments (A–F).
**Figure S3.** Scatter plot of forb richness in the six herbivory treatments (A–F).
**Figure S4.** Scatter plot of grass richness in six herbivory treatments (A–F).
**Figure S5.** Scatter plot of mean bare soil cover (natural‐log‐transformed) in six herbivory treatments (A–F).
**Figure S6.** Scatter plot of tree density (ha) in six herbivory treatments (A–F).
**Figure S7.** Principal component analysis of five vegetation variables measured at six herbivory treatments (A–F).
**Figure S8.** Scatter plot of (a) abundance and (b) order richness of invertebrates in six herbivory treatments (A–F).
**Figure S9.** Scatter plot of Orthoptera abundance in six herbivory treatments (A–F).

## Data Availability

Raw data are available from the Dryad Digital Repository https://doi.org/10.5061/dryad.kwh70rzk2 (Matthies et al., 2026).
