# Supplementary material for: Mammalian herbivory indirectly shapes savanna arthropod communities but only at very low or high levels
Source: J Anim Ecol. 2026 Jan 28;95(4):658–71. doi: 10.1111/1365-2656.70221 (PMC13039251; doi:10.1111/1365-2656.70221)
Supplement: Supplementary file 1 — Table S1. Description of the study landscape and distribution of large mammalian herbivory treatments. Table S2. Numbers of large mammalian herbivores counted in Lewa and Borana 2022–2024 (Game Count Report Lewa Borana 2024). Table S3. List of invertebrate orders found across the 20 sampling plots, with total abundance across all plots. Table S4. Ant abundance and species richness for each of the 20 sampling plots across six treatments (A–F, see Table 1 for descriptions of treatments). Table S5. Tukey HSD post‐hoc pair‐wise comparisons between six treatments (A–F) for ant species richness (natural‐log‐transformed). Table S6. Tukey HSD post‐hoc pair‐wise comparisons between six treatments (A–F) for the environmental variables: mean height of herbaceous layer, grass richness (natural‐log‐transformed) and mean bare soil (natural‐log‐transformed). Table S7. Dunn's test pair‐wise comparisons between six treatments (A–F) for tree density using Benjamini–Hochberg correction. Figure S1. Species accumulation curves for the six herbivory treatments (A–F). Figure S2. Scatter plot of mean height (cm) of herbaceous vegetation in the six herbivory treatments (A–F). Figure S3. Scatter plot of forb richness in the six herbivory treatments (A–F). Figure S4. Scatter plot of grass richness in six herbivory treatments (A–F). Figure S5. Scatter plot of mean bare soil cover (natural‐log‐transformed) in six herbivory treatments (A–F). Figure S6. Scatter plot of tree density (ha) in six herbivory treatments (A–F). Figure S7. Principal component analysis of five vegetation variables measured at six herbivory treatments (A–F). Figure S8. Scatter plot of (a) abundance and (b) order richness of invertebrates in six herbivory treatments (A–F). Figure S9. Scatter plot of Orthoptera abundance in six herbivory treatments (A–F). [file JANE-95-658-s001.docx]

**Supplementary Information:**

**Mammalian herbivory indirectly shapes savanna arthropod communities, but only at very low or high levels**

**Table S1.** Description of the study landscape and distribution of large mammalian herbivory treatments

**Table S2.** Numbers of large mammalian herbivores counted in Lewa and Borana 2022 – 2024 (Game Count Report Lewa Borana 2024)*.* Livestock in Lewa and Borana consists of cattle, goats, sheep and camels.

**Table S3.** List of invertebrate orders found across the 20 sampling plots, with total abundance across all plots.

**Table S4.** Ant abundance and species richness for each of the 20 sampling plots across six treatments (A to F, see Table 1 for descriptions of treatments).

**Table S5.** Tukey HSD post-hoc pair-wise comparisons between six treatments (A to F) for ant species richness (natural-log-transformed).

**Table S6.** Tukey HSD post-hoc pair-wise comparisons between six treatments (A to F) for the environmental variables: mean height of herbaceous layer, grass richness (natural-log-transformed) and mean bare soil (natural-log-transformed).

**Table S7.** Dunn's Test pair-wise comparisons between six treatments (A to F) for tree density using Benjamini-Hochberg correction.

**Figure S1.** Figure S1: Species accumulation curves for the six herbivory treatments (A to F). Ant species richness (q = 0 diversity) per treatment was computed as the mean of 200 bootstrap replicates with 95% confidence intervals. The y-axis represents species richness, and the x-axis represents the sampling effort (number of pitfall traps). The solid lines represent the interpolated species richness, the dotted lines the extrapolated species richness with 95% confidence intervals. The species richness was extrapolated for each treatment, i.e. double the amount of recorded pitfall traps used per treatment in the study (45-60).

**Figure S2.** Scatter plot of mean height (cm) of herbaceous vegetation in the six herbivory treatments A to F. Letters indicate statistically significant differences between treatments, treatments sharing the same letter are not significantly different. Each point represents a plot.

**Figure S3.** Scatter plot of forb richness in the six herbivory treatments A to F. Each point represents a plot.

**Figure S4.** Scatter plot of grass richness in six herbivory treatments A to F. Letters indicate statistically significant differences between treatments, treatments sharing the same letter are not significantly different. Each point represents a plot.

**Figure S5.** Scatter plot of mean bare soil cover (natural-log-transformed) in six herbivory treatments A to F. Letters indicate statistically significant differences between treatments, treatments sharing the same letter are not significantly different. Each point represents a plot.

**Figure S6.** Scatter plot of tree density (ha) in six herbivory treatments A to F. Letters indicate statistically significant differences between treatments, treatments sharing the same letter are not significantly different. Each point represents a plot.

**Figure S7.** Principal Component Analysis of five vegetation variables measured at six herbivory treatments (A to F). Arrows indicate the five vegetation variables: Bare ground, Forb richness, Grass richness, Herbaceous vegetation height and Tree density. Each point represents a plot (n = 18).

**Figure S8a** and **b:** Scatter plot of a) abundance and b) order richness of invertebrates in six herbivory treatments A to F. Abundance and Order richness did not differ significantly across herbivory systems (ANOVA Abundance: F₅,₁₄ = 2.607, p = 0.072; ANOVA Order richness; F₅,₁₄ = 2.52, p = 0.079).

**Figure S9:** Scatter plot of Orthoptera abundance in six herbivory treatments A to F. Orthoptera abundance (log-transformed) differed significantly among herbivory treatments (ANOVA; F₅,₁₄ = 17.15, p < 0.001). Letters indicate statistically significant differences (Tukey HSD, p < 0.05) between treatments, treatments sharing the same letter are not significantly different. Each point represents a plot.

Table S1– Distribution of large mammalian herbivory treatments across the study landscape (Lewa Wildlife Conservancy and Borana Conservancy) and detailed description of the study landscape.

| **Conservancy** | **Description** | **Treatments present** |
| --- | --- | --- |
| Lewa Wildlife Conservancy | Lewa Wildlife Conservancy is a 20,000 ha former cattle ranch that has been transformed into a privately managed wildlife conservation area (**C**). The conservancy is used for photographic tourism but allows unrestricted livestock grazing along a community road that crosses the conservancy (**A**). Elephant and large mammalian exclusion zones have been used as a major management intervention over the last 30 years to increase the tree cover, with numerous exclusion zones of different age categories (**D, E**). | **(A) Wildlife and high intensity livestock**  **(C) Wildlife**  **(D) Wildlife – No megaherbivores (young)**  **(E) Wildlife – No megaherbivores (old)** |
| Borana Conservancy | Borana conservancy is a 12,000 ha dual-use wildlife and livestock ranch. This conservancy is used for photographic tourism but also produces cattle and extends limiting grazing rights to local community herders, which mainly rely on rotational grazing (**B**). Full mammalian exclusion zones have been used since 1970 to increase the tree cover, and have been recently refenced in 2023 (**F**). | **(B) Wildlife and livestock**  **(F) Full exclosure** |

Table S2: Numbers of large mammalian herbivores counted in Lewa and Borana 2022 – 2024 (Game Count Report Lewa Borana 2024)*.* Livestock in Lewa and Borana consists of cattle, goats, sheep and camels.

| **Species** | **2022** | **2023** | **2024** |
| --- | --- | --- | --- |
| *Aepyceros melampus* | 1285 | 1523 | 1692 |
| *Alcelaphus buselaphus* | 92 | 199 | 73 |
| *Ceratotherium simum* | 118 | 123 | 123 |
| *Diceros bicornis* | 132 | 133 | 120 |
| *Equus grevyi* | 310 | 359 | 332 |
| *Equus quagga burchellii* | 1557 | 1731 | 1382 |
| *Eudorcas thomsonii* | 26 | 4 | 6 |
| *Giraffa camelopardalis reticulata* | 119 | 138 | 123 |
| *Hippopotamus amphibius* | 2 | 2 | 2 |
| *Kobus ellipsiprymnus* | 158 | 224 | 109 |
| *Litocranius walleri* | 16 | 12 | 20 |
| *Loxodonta africana* | 425 | 356 | 499 |
| *Nanger granti* | 1135 | 1210 | 1316 |
| *Oreotragus oreotragus* | 15 | 13 | 13 |
| *Oryx beisa* | 239 | 285 | 214 |
| *Phacochoerus africanus* | 151 | 157 | 81 |
| *Syncerus caffer* | 1901 | 715 | 512 |
| *Taurotragus oryx* | 331 | 245 | 192 |
| *Tragelaphus strepsiceros* | 32 | 29 | 37 |
| *Tragelaphus sylvaticus* | 17 | 19 | 8 |

Table S3: List of invertebrate orders found across the 20 sampling plots, with total abundance across all plots.

| Invertebrate Order | Abundance | | |
| --- | --- | --- | --- |
| Collembola | | 2286 |  |
| Hemiptera | | 1092 |  |
| Diptera | | 1040 |  |
| Coleoptera | | 847 |  |
| Araneae | | 558 |  |
| Hymenoptera *(excluding ants)* | | 429 |  |
| Acari | | 421 |  |
| Orthoptera | | 363 |  |
| Lepidoptera_larvae | | 110 |  |
| Blattodea | | 21 |  |
| Psocoptera | | 13 |  |
| Chilopoda | | 11 |  |
| Lepidoptera | | 9 |  |
| Thysanoptera | | 8 |  |
| Isoptera | | 7 |  |
| Opiliones | | 4 |  |
| Solifugae | | 4 |  |
| Isopoda | | 3 |  |
| Mantodea | | 3 |  |
| Pseudoscorpiones | | 2 |  |
| Siphonaptera | | 2 |  |
| Diplopoda | | 1 |  |

Table S4: Ant abundance and species richness for each of the 20 sampling plots across six treatments (A to F, see Table 1 for descriptions of treatments).

| Plot | Treatment | Richness | Abundance |
| --- | --- | --- | --- |
| 1 | A | 23 | 520 |
| 2 | A | 7 | 88 |
| 3 | A | 23 | 560 |
| 4 | A | 10 | 49 |
| 5 | B | 5 | 157 |
| 6 | B | 9 | 646 |
| 7 | B | 6 | 1,291 |
| 8 | C | 26 | 327 |
| 9 | C | 14 | 447 |
| 10 | C | 10 | 72 |
| 11 | C | 18 | 602 |
| 12 | D | 11 | 1,282 |
| 13 | D | 9 | 678 |
| 14 | D | 10 | 43 |
| 15 | E | 22 | 281 |
| 16 | E | 10 | 34 |
| 17 | E | 16 | 1,379 |
| 18 | F | 10 | 490 |
| 19 | F | 7 | 245 |
| 20 | F | 5 | 687 |

Table S5: Tukey HSD post-hoc pair-wise comparisons between six treatments (A to F) for ant species richness (natural-log-transformed).

| Comparison | Difference | Lower_CI | Upper_CI | Adjusted_p |
| --- | --- | --- | --- | --- |
| B-A | -0.764 | -1.786 | 0.258 | 0.204 |
| C-A | 0.143 | -0.804 | 1.089 | 0.996 |
| D-A | -0.331 | -1.353 | 0.691 | 0.889 |
| E-A | 0.092 | -0.930 | 1.114 | 1.000 |
| F-A | -0.677 | -1.699 | 0.345 | 0.308 |
| C-B | 0.906 | -0.116 | 1.929 | 0.096 |
| D-B | 0.433 | -0.660 | 1.526 | 0.780 |
| E-B | 0.856 | -0.237 | 1.949 | 0.169 |
| F-B | 0.087 | -1.006 | 1.179 | 1.000 |
| D-C | -0.473 | -1.495 | 0.549 | 0.659 |
| E-C | -0.050 | -1.073 | 0.972 | 1.000 |
| F-C | -0.820 | -1.842 | 0.202 | 0.153 |
| E-D | 0.423 | -0.670 | 1.516 | 0.796 |
| F-D | -0.347 | -1.439 | 0.746 | 0.896 |
| F-E | -0.769 | -1.862 | 0.323 | 0.253 |
|  | | | | |

Table S6: Tukey HSD post-hoc pair-wise comparisons between six treatments (A to F) for the environmental variables: mean height of herbaceous layer, grass richness (natural-log-transformed) and mean bare soil (natural-log-transformed).

| Response Variable | Comparison | Difference | 95% CI Lower | 95% CI Upper | Adj. p-value |
| --- | --- | --- | --- | --- | --- |
| Mean Height of  Herbaceous Layer | B-A | 12.872 | -0.020 | 25.764 | **0.050** |
|  | C-A | 13.943 | 1.051 | 26.835 | **0.032** |
|  | D-A | 16.067 | 3.175 | 28.959 | **0.012** |
|  | E-A | 13.178 | 0.286 | 26.070 | **0.044** |
|  | F-A | 9.373 | -3.519 | 22.265 | 0.216 |
|  | C-B | 1.071 | -11.821 | 13.963 | 1.000 |
|  | D-B | 3.195 | -9.697 | 16.087 | 0.955 |
|  | E-B | 0.306 | -12.586 | 13.198 | 1.000 |
|  | F-B | -3.498 | -16.390 | 9.394 | 0.936 |
|  | D-C | 2.124 | -10.768 | 15.016 | 0.992 |
|  | E-C | -0.765 | -13.657 | 12.127 | 1.000 |
|  | F-C | -4.569 | -17.461 | 8.323 | 0.833 |
|  | E-D | -2.889 | -15.781 | 10.003 | 0.971 |
|  | F-D | -6.693 | -19.585 | 6.199 | 0.531 |
|  | F-E | -3.805 | -16.697 | 9.087 | 0.912 |
|  | | | | | |
| Grass Richness (log) | B-A | -0.837 | -1.746 | 0.072 | 0.078 |
|  | C-A | -0.580 | -1.489 | 0.328 | 0.328 |
|  | D-A | -0.456 | -1.365 | 0.453 | 0.564 |
|  | E-A | -0.391 | -1.300 | 0.517 | 0.701 |
|  | F-A | -1.348 | -2.257 | -0.439 | **0.003** |
|  | C-B | 0.257 | -0.652 | 1.166 | 0.926 |
|  | D-B | 0.381 | -0.528 | 1.290 | 0.722 |
|  | E-B | 0.446 | -0.463 | 1.355 | 0.586 |
|  | F-B | -0.511 | -1.420 | 0.398 | 0.453 |
|  | D-C | 0.124 | -0.785 | 1.033 | 0.997 |
|  | E-C | 0.189 | -0.720 | 1.098 | 0.979 |
|  | F-C | -0.768 | -1.676 | 0.141 | 0.118 |
|  | E-D | 0.065 | -0.844 | 0.974 | 1.000 |
|  | F-D | -0.892 | -1.801 | 0.017 | 0.056 |
|  | F-E | -0.957 | -1.865 | -0.048 | **0.037** |
|  | | | | | |
| Mean Bare  Soil (log) | B-A | -1.975 | -3.473 | -0.477 | **0.008** |
|  | C-A | -0.381 | -1.879 | 1.117 | 0.951 |
|  | D-A | -1.293 | -2.791 | 0.205 | 0.107 |
|  | E-A | -0.928 | -2.426 | 0.570 | 0.357 |
|  | F-A | -0.883 | -2.381 | 0.615 | 0.406 |
|  | C-B | 1.594 | 0.096 | 3.092 | **0.035** |
|  | D-B | 0.682 | -0.816 | 2.180 | 0.654 |
|  | E-B | 1.047 | -0.451 | 2.545 | 0.248 |
|  | F-B | 1.092 | -0.406 | 2.590 | 0.214 |
|  | D-C | -0.912 | -2.410 | 0.586 | 0.374 |
|  | E-C | -0.547 | -2.045 | 0.951 | 0.816 |
|  | F-C | -0.502 | -2.000 | 0.996 | 0.862 |
|  | E-D | 0.365 | -1.133 | 1.863 | 0.959 |
|  | F-D | 0.410 | -1.088 | 1.908 | 0.934 |
|  | F-E | 0.046 | -1.452 | 1.543 | 1.000 |

Table S7: Dunn's Test pair-wise comparisons between six treatments (A to F) for tree density using Benjamini-Hochberg correction.

| Comparison | P_Value | Adjusted_P_Value | Effect_Size |
| --- | --- | --- | --- |
| A - B | 0.500 | 0.500 | 0 |
| A - C | 0.326 | 0.350 | -0.450 |
| B - C | 0.326 | 0.377 | -0.450 |
| A - D | 0.153 | 0.230 | -1.022 |
| B - D | 0.153 | 0.256 | -1.022 |
| C - D | 0.284 | 0.354 | -0.572 |
| A - E | 0.014 | **0.041** | -2.207 |
| B - E | 0.014 | **0.051** | -2.207 |
| C - E | 0.039 | 0.084 | -1.758 |
| D - E | 0.118 | 0.221 | -1.185 |
| A - F | 0.002 | **0.012** | -2.943 |
| B - F | 0.002 | 0.024 | -2.943 |
| C - F | 0.006 | **0.032** | -2.493 |
| D - F | 0.027 | 0.068 | -1.921 |
| E - F | 0.231 | 0.315 | -0.736 |


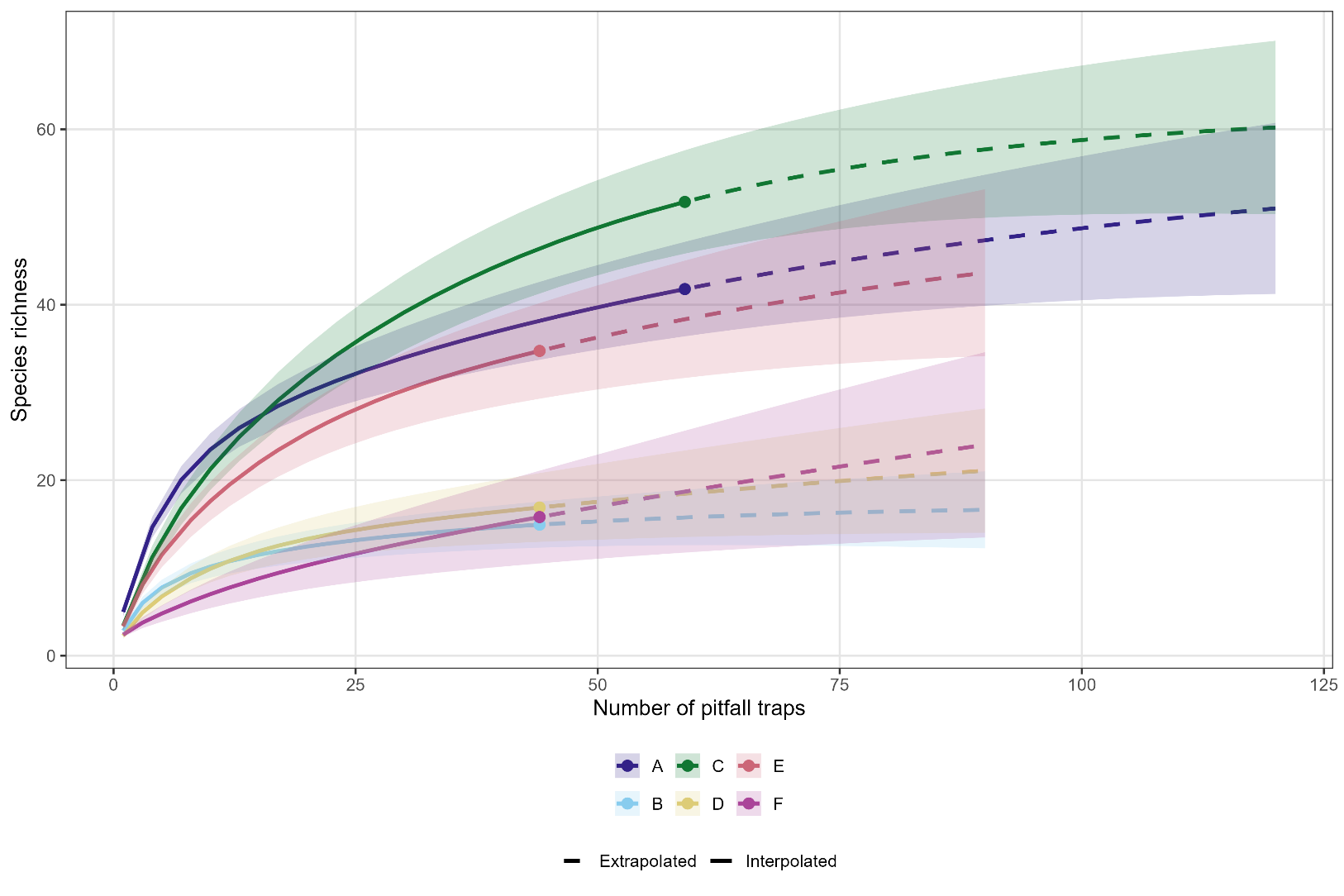


Figure S1: Species accumulation curves for the six herbivory treatments A to F. Ant species richness (q = 0 diversity) per treatment was computed as the mean of 200 bootstrap replicates with 95% confidence intervals. The y-axis represents species richness, and the x-axis represents the sampling effort (number of pitfall traps). The solid lines represent the interpolated species richness, the dotted lines the extrapolated species richness with 95% confidence intervals. The species richness was extrapolated for each treatment, i.e. double the amount of recorded pitfall traps used per treatment in the study (45-60).


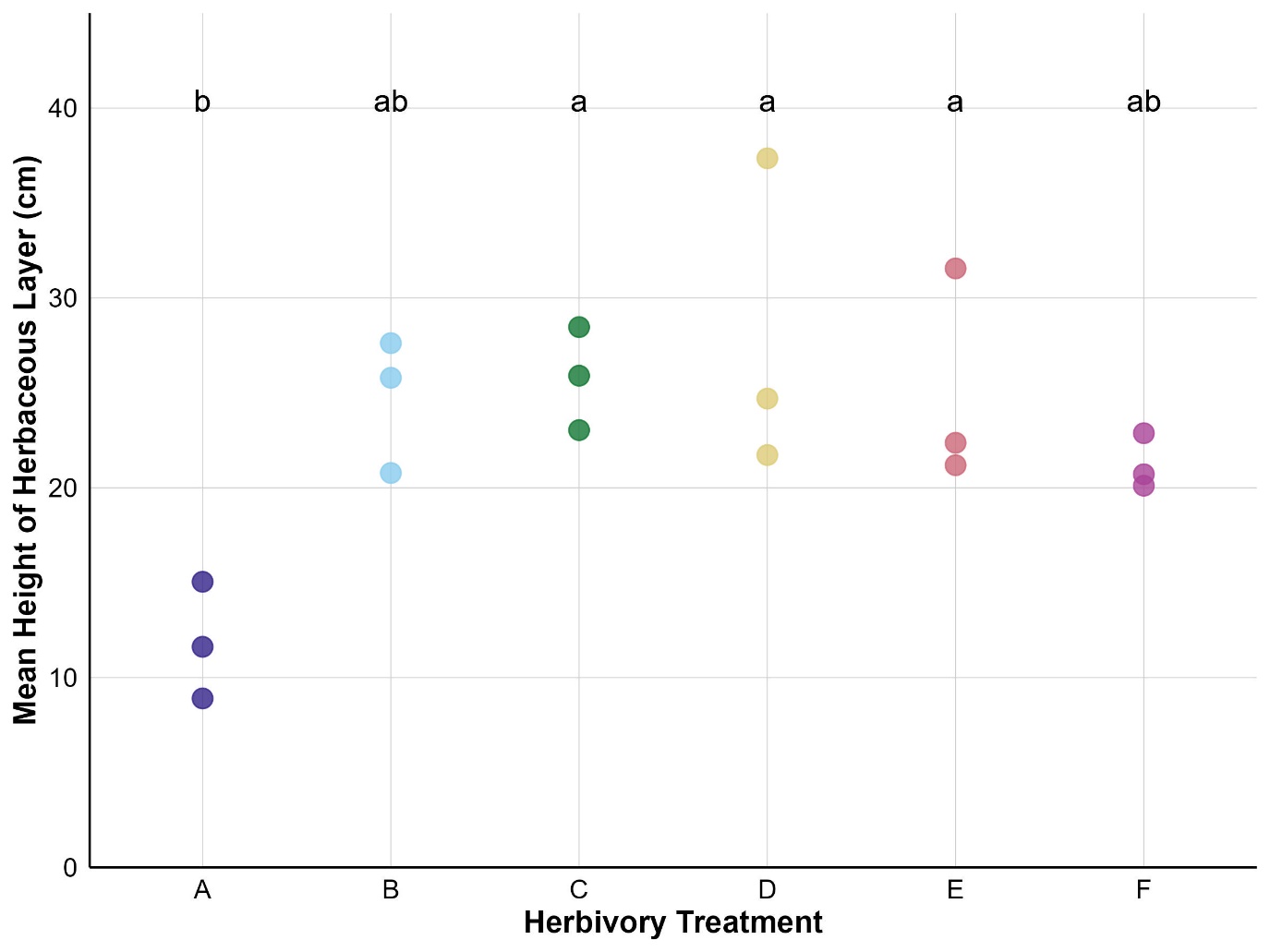


Figure S2: Scatter plot of mean height (cm) of herbaceous vegetation in the six herbivory treatments A to F. Letters indicate statistically significant differences between treatments, treatments sharing the same letter are not significantly different. Each point represents a plot.


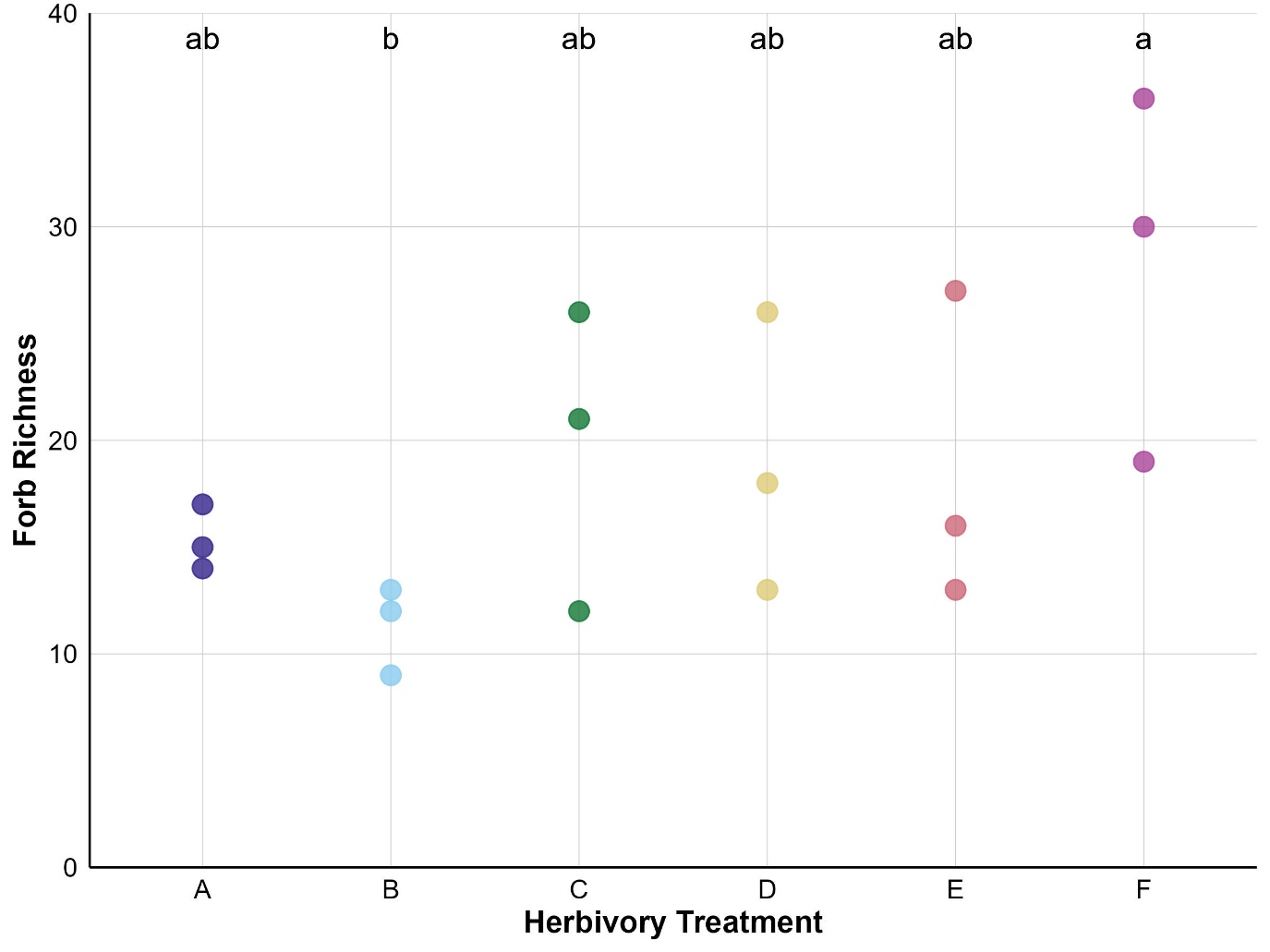


Figure S3: Scatter plot of forb richness in the six herbivory treatments A to F. Each point represents a plot.


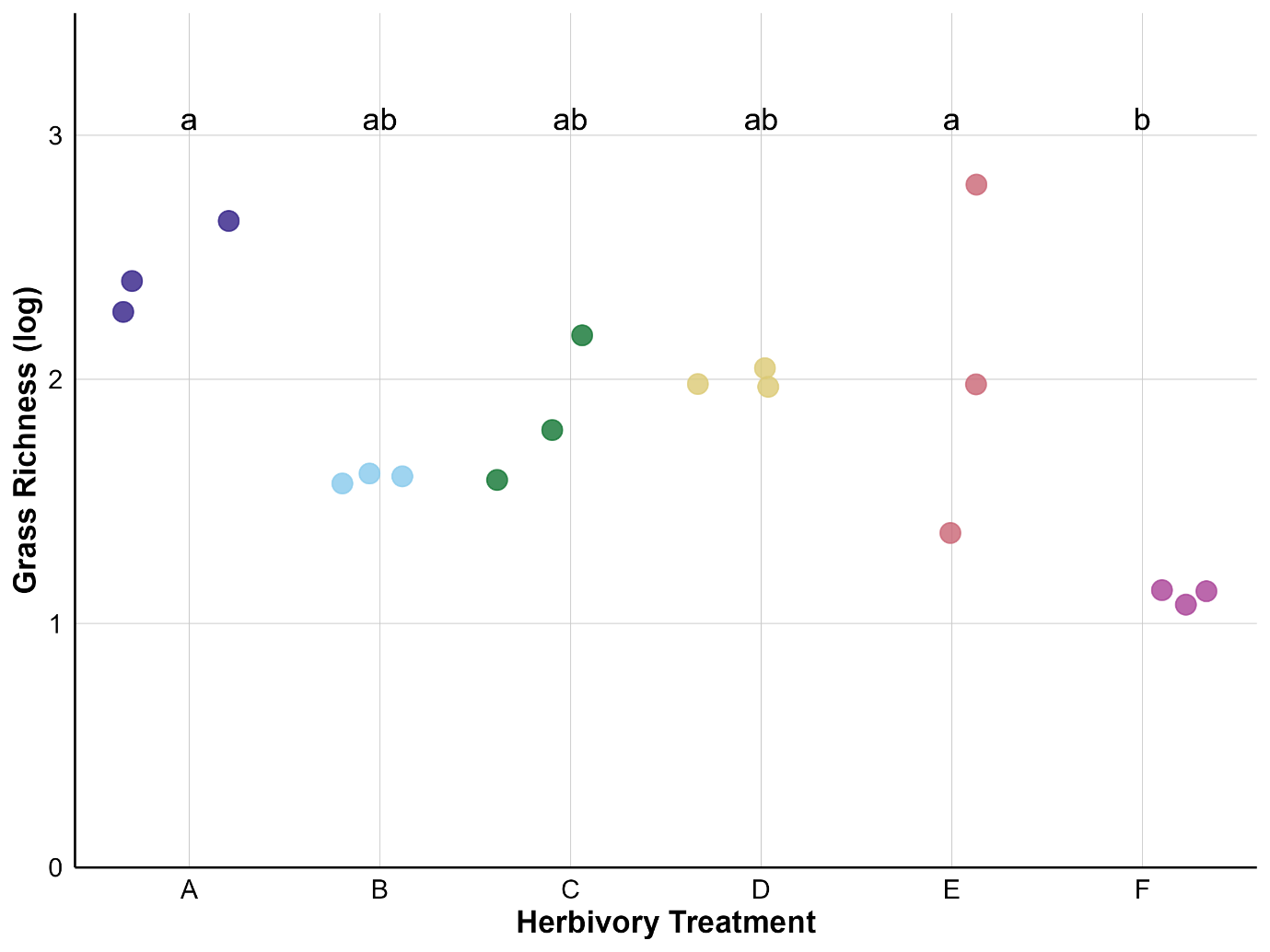


Figure S4: Scatter plot of grass richness in six herbivory treatments A to F. Letters indicate statistically significant differences between treatments, treatments sharing the same letter are not significantly different. Each point represents a plot.


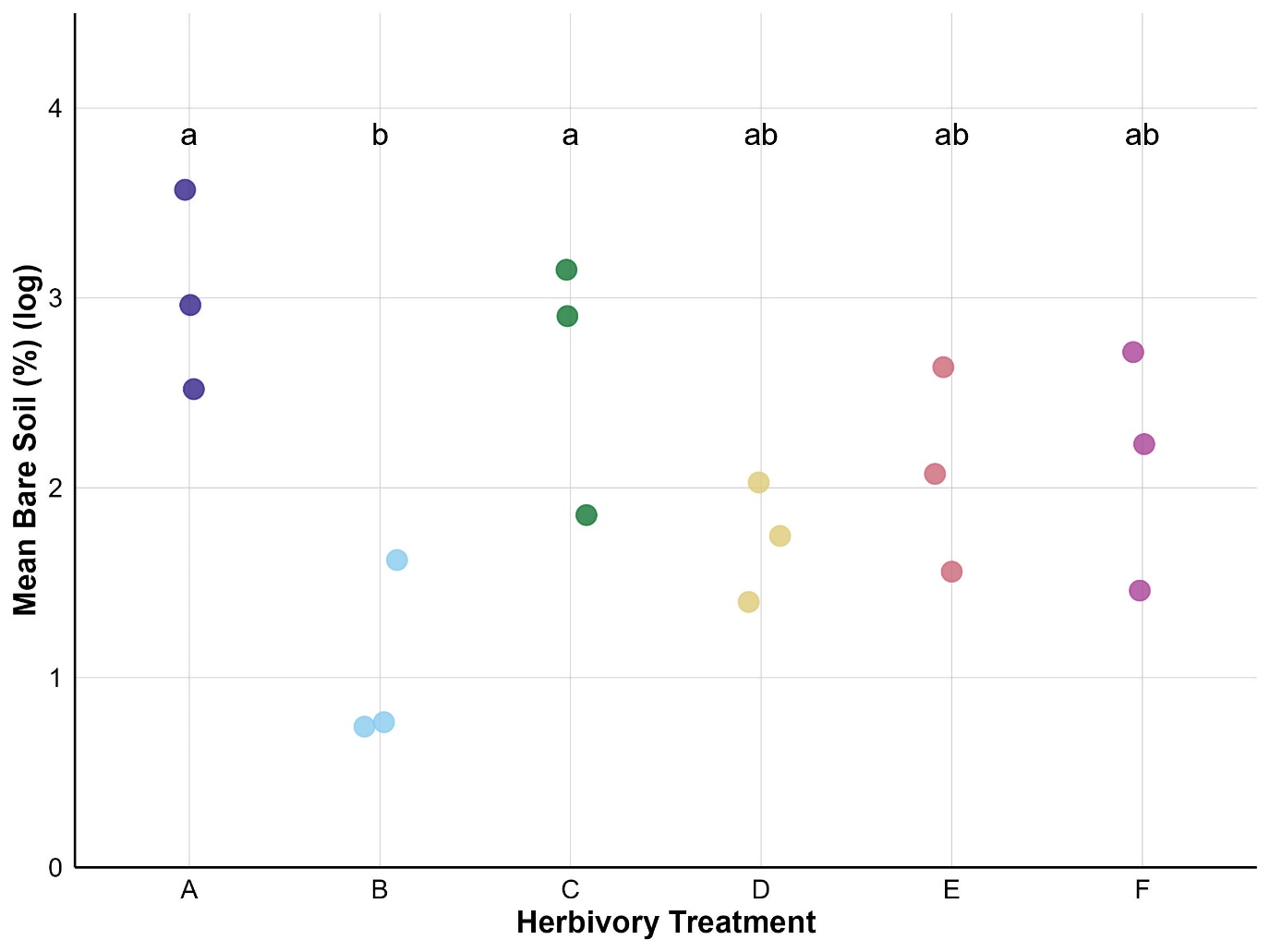


Figure S5: Scatter plot of mean bare soil cover (natural-log-transformed) in six herbivory treatments A to F. Letters indicate statistically significant differences between treatments, treatments sharing the same letter are not significantly different. Each point represents a plot.


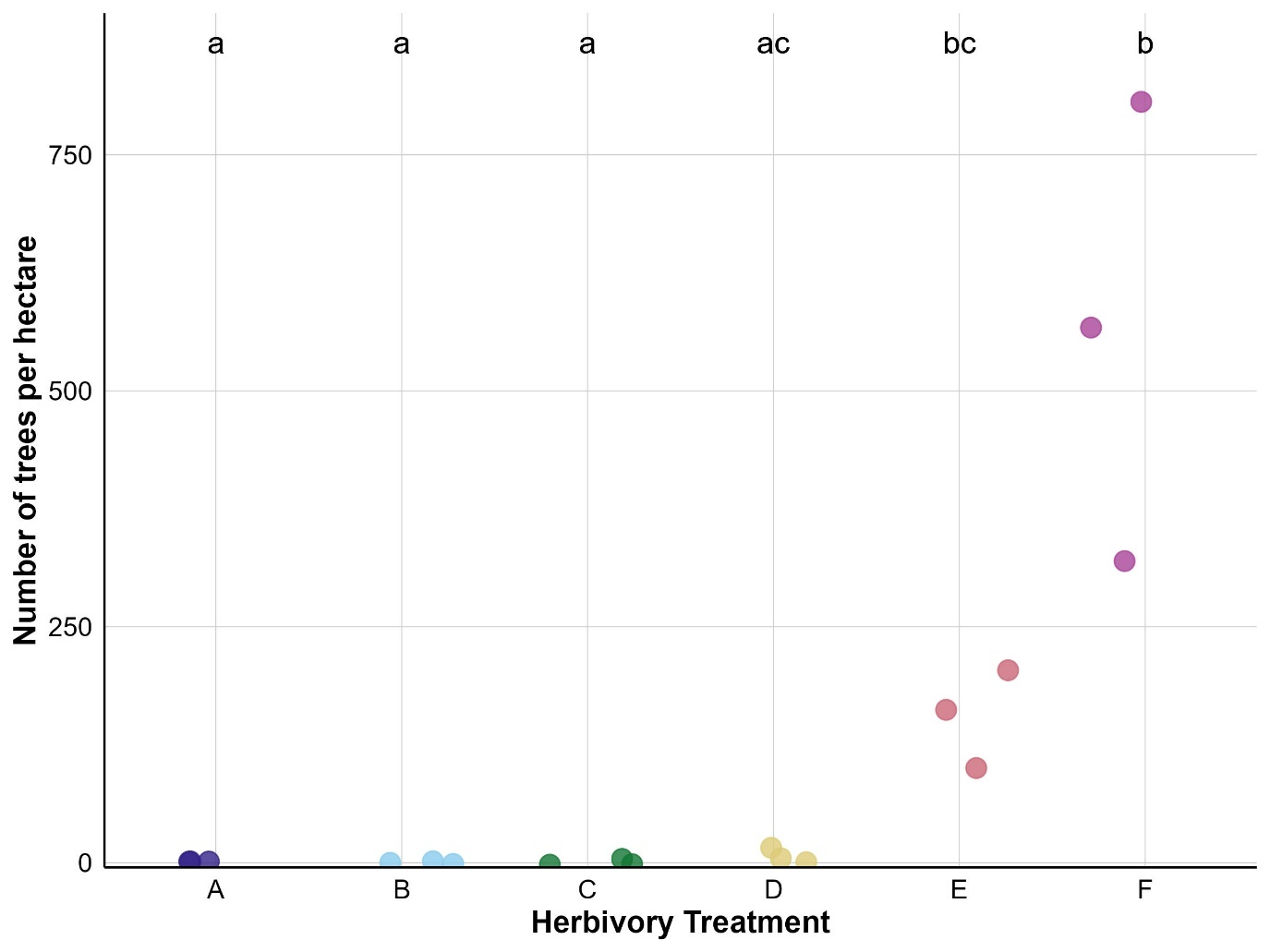


Figure S 6: Scatter plot of tree density (number of trees per hectare) in six herbivory treatments A to F. Letters indicate statistically significant differences between treatments, treatments sharing the same letter are not significantly different. Each point represents a plot.


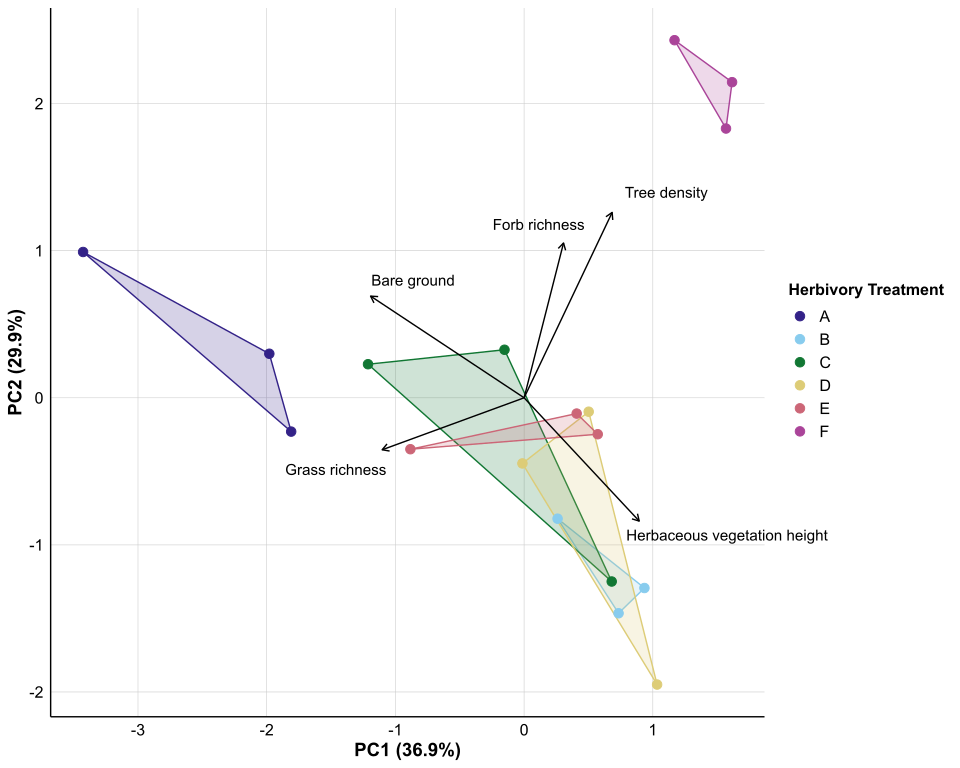
 Figure S7: Principal Component Analysis of five vegetation variables measured at six herbivory treatments (A to F). Arrows indicate the five vegetation variables: Bare ground, Forb richness, Grass richness, Herbaceous vegetation height and Tree density. Each point represents a plot (n = 18).


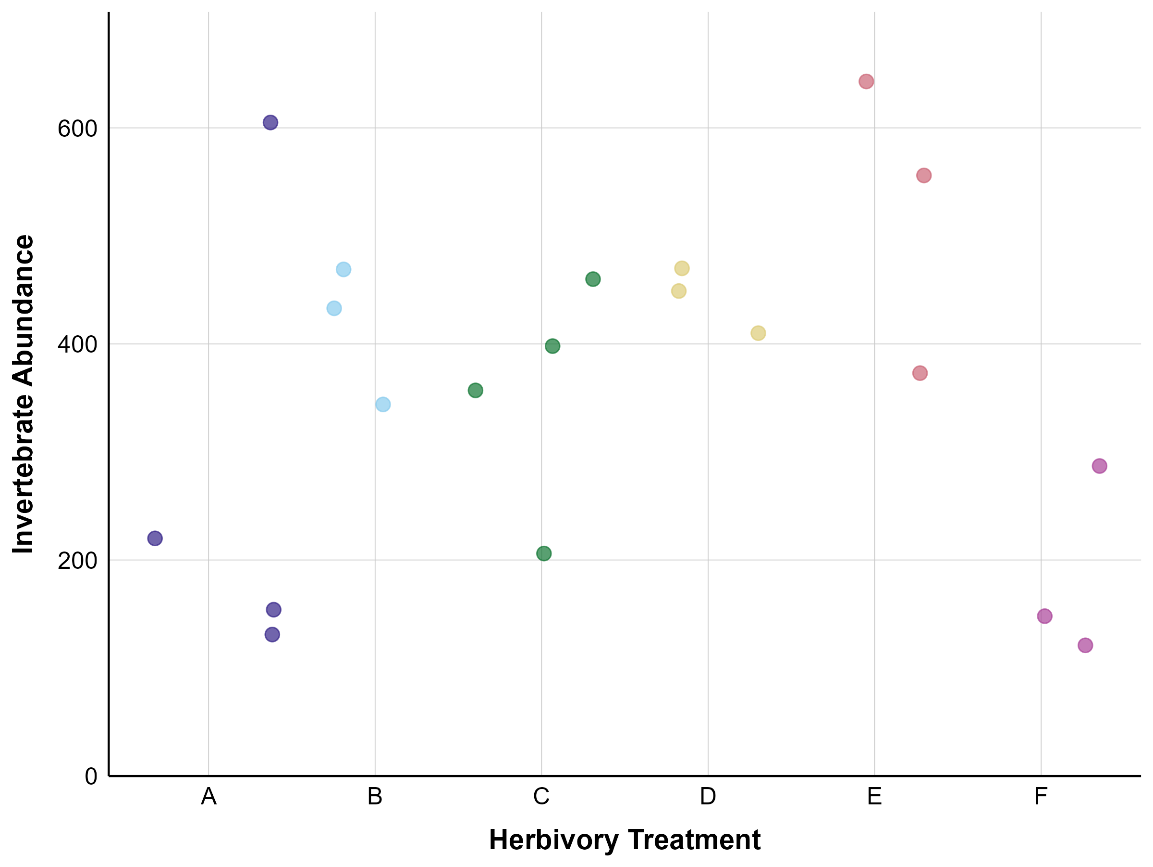

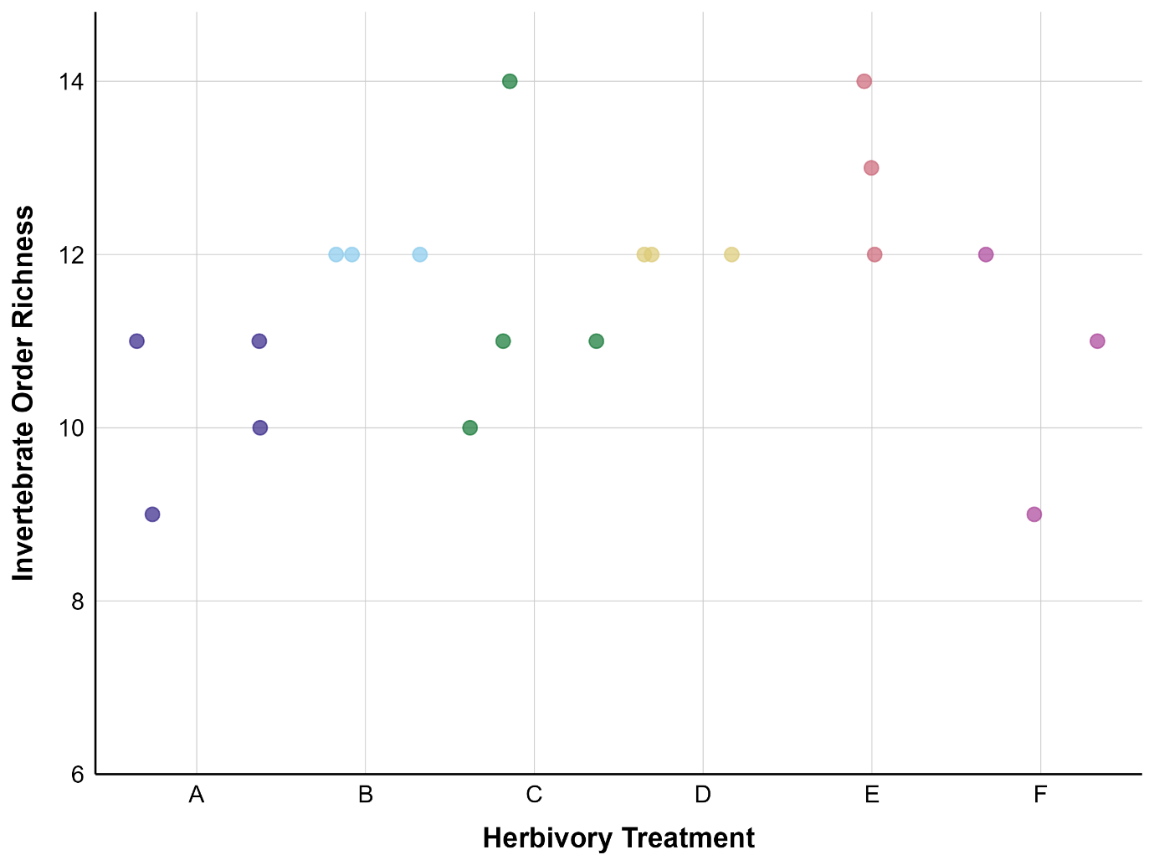


**b)**

**a)**

Figure S8a and b: Scatter plot of a) abundance and b) order richness of invertebrates in six herbivory treatments A to F. Abundance and order richness did not differ significantly across herbivory systems (ANOVA Abundance: F₅,₁₄ = 2.607, p = 0.072; ANOVA Order richness; F₅,₁₄ = 2.52, p = 0.079).


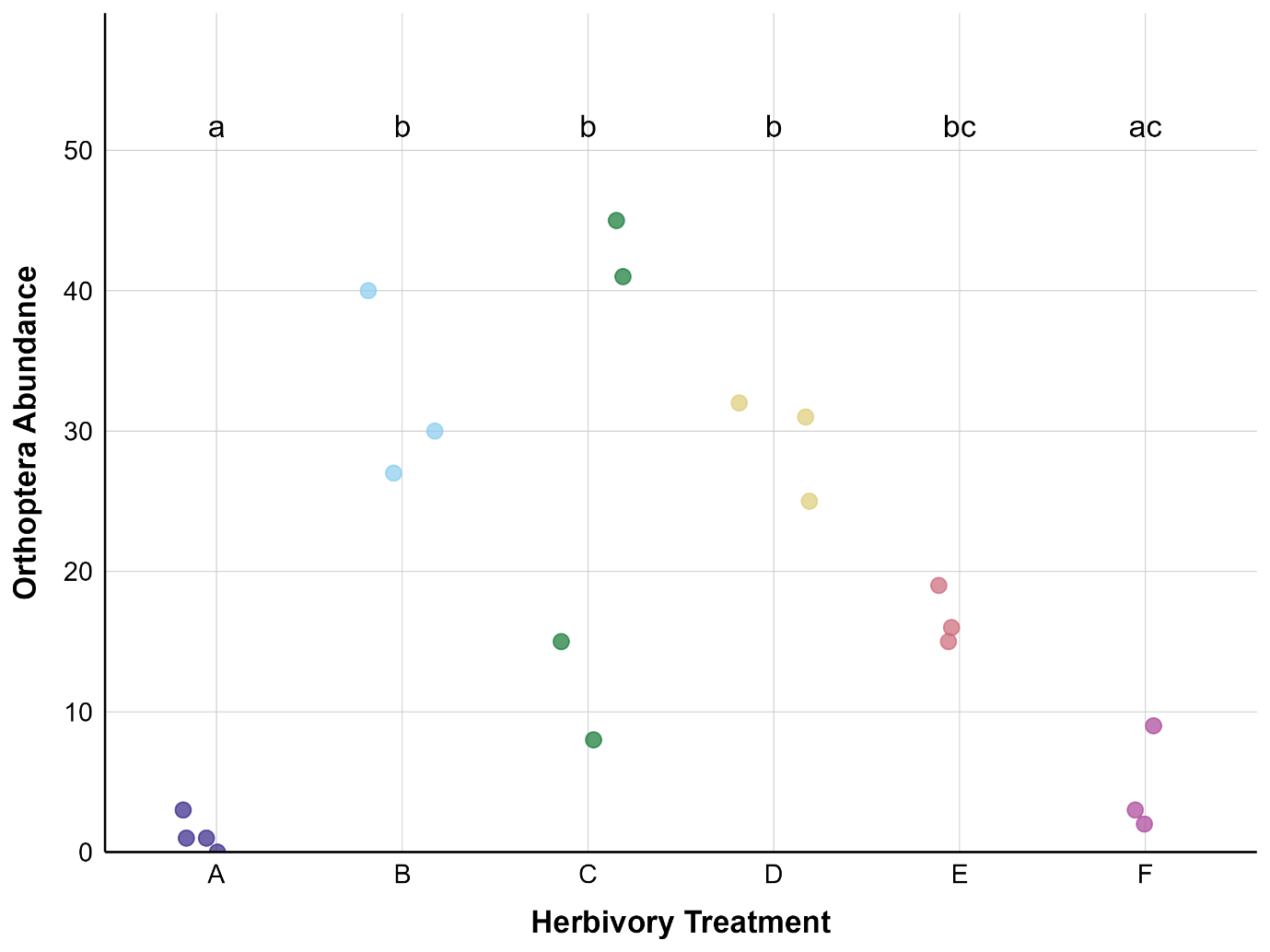


Figure S9: Scatter plot of Orthoptera abundance in six herbivory treatments A to F. Orthoptera abundance (log-transformed) differed significantly among herbivory treatments (ANOVA; F₅,₁₄ = 17.15, p < 0.001). Letters indicate statistically significant differences (Tukey HSD, p < 0.05) between treatments, treatments sharing the same letter are not significantly different. Each point represents a plot.
